# Bayesian linear mixed model with multiple random effects for prediction analysis on high-dimensional multi-omics data

**DOI:** 10.1093/bioinformatics/btad647

**Published:** 2023-10-26

**Authors:** Yang Hai, Jixiang Ma, Kaixin Yang, Yalu Wen

**Affiliations:** Department of Health Statistics, Shanxi Medical University, Taiyuan, Shanxi Province 030000, China; Department of Statistics, University of Auckland, Auckland 1010, New Zealand; Department of Health Statistics, Shanxi Medical University, Taiyuan, Shanxi Province 030000, China; Department of Health Statistics, Shanxi Medical University, Taiyuan, Shanxi Province 030000, China; Department of Health Statistics, Shanxi Medical University, Taiyuan, Shanxi Province 030000, China; Department of Statistics, University of Auckland, Auckland 1010, New Zealand

## Abstract

**Motivation:**

Accurate disease risk prediction is an essential step in the modern quest for precision medicine. While high-dimensional multi-omics data have provided unprecedented data resources for prediction studies, their high-dimensionality and complex inter/intra-relationships have posed significant analytical challenges.

**Results:**

We proposed a two-step Bayesian linear mixed model framework (TBLMM) for risk prediction analysis on multi-omics data. TBLMM models the predictive effects from multi-omics data using a hybrid of the sparsity regression and linear mixed model with multiple random effects. It can resemble the shape of the true effect size distributions and accounts for non-linear, including interaction effects, among multi-omics data via kernel fusion. It infers its parameters via a computationally efficient variational Bayes algorithm. Through extensive simulation studies and the prediction analyses on the positron emission tomography imaging outcomes using data obtained from the Alzheimer’s Disease Neuroimaging Initiative, we have demonstrated that TBLMM can consistently outperform the existing method in predicting the risk of complex traits.

**Availability and implementation:**

The corresponding R package is available on GitHub (https://github.com/YaluWen/TBLMM).

## 1 Introduction

Emerging high-dimensional multi-omics data hold great promise in precision medicine, as they have the potentials in capturing comprehensive profiles of samples at multiple levels, including genomics, transcriptomics, epigenomics, and interactomics (Huang *et al.* 2017, Kornej *et al.* 2021, Guan *et al.* 2022). Although the single-omics analysis has been partially successful in the previous omics studies (Yang *et al.* 2010), it has recently become clear that single-omics data has only provided limited information regarding the complexity of biological systems (Gygi *et al.* 1999, Huang *et al.* 2017). For example, ter Kuile and Westerhoff (2001) has shown glycolysis is not only regulated by gene expression but also the control of glycolysis could be shared at metabolic, proteomic and genomic levels. So far, the role of various omics data and their underlying mechanisms in mediating outcome of human diseases remained largely unknown. An efficient framework of integrating multi-omics is needed to enhance the understanding of their functions and facilitate precision medicine (Huang *et al.* 2017, Olivier *et al.* 2019).

Tremendous efforts have been made to analyze and interpret omics data in recent years (Vaske *et al.* 2010, Shen *et al.* 2013, Angione *et al.* 2016, Graw *et al.* 2021). One of the common goal of omics integration is to detect molecular patterns. For example, PARADIGM (Vaske *et al.* 2010), a factor graph framework, was developed to integrate multiple omics and functional omics datasets for pathway inference. Akavia *et al.* (2010) proposed Conexic algorithm to integrate gene expression and copy number data in detecting mutation drivers. Multi-block Partial Least Squares (Li *et al.* 2012) first summaries each layer of the dataset as a latent variable and then integrates them to detect common patterns. Another main objective of omics integration is to understand the partition of samples based on the underlying molecular patterns. For example, Shen *et al.* (2013) proposed the parametric Bayesian-based method iCluster to integrate multi-omics data that can be both continuous and categorical for classification (Bersanelli *et al.* 2016, Zeng and Lumley 2018). MOGONET (Wang *et al.* 2021) has been proposed to reveal similarities between two classes of samples, where graph convolutional networks are first built for each omics layer and then integrated into one network with View Correlation Discovery Network. Despite current advancements, there are still few methods available that can efficiently integrate multi-omics data for risk prediction on complex continuous traits (Bersanelli *et al.* 2016).

The integration of multi-omics data can be computationally intensive due to their high-dimensional nature (Schumacher *et al.* 2014, Miao *et al.* 2021), and dimension reduction becomes an indispensable step (Meng *et al.* 2016). Principal components analysis (PCA), partial least squares (PLS), low-rank approximation (LRA), and Canonical correlation analysis (CCA) are widely used for selecting important variables from high-dimensional omics data (Bersanelli *et al.* 2016, Zeng and Lumley 2018). For example, Multi-block PCA is used to identify common information from multi-omics data (Li *et al.* 2012, Satagopam *et al.* 2016, Zeng and Lumley 2018). Multi-block PLS reduces the data dimension by first summarizing each layer of omics data as a latent variable and then assigning weights to each omics layer, which are obtained by maximizing variance between the input and response. LRA concatenates all omics layers into a single matrix and uses low-rank approximation to reduce the dimension (Wu *et al.* 2015). CCA reduces the data dimension by finding projections for each of omics data, where projected data maximize correlations (Härdle and Simar 2015). Sparse CCA can further reduce the number of selected variables via penalization (Hardoon and Shawe-Taylor 2011, Rodosthenous *et al.* 2020). While existing dimension reduction methods can facilitate the downstream analysis of multi-omics data, they are not designed to detect variables that maximize the prediction accuracy. As such, important predictors can be overlooked, leading to sub-optimal prediction performance.

Linear mixed model and its extensions (LMMs) are widely used tools for prediction analysis with genomic data (De los Campos *et al.* 2013, Speed and Balding 2014, Weissbrod *et al.* 2016, Wen and Lu 2020, Wang and Wen 2022a), and they have also been extended for omics data analyses (Li *et al.* 2020, Wang and Wen 2022b). LMMs assume that similarities in omics can lead to similarities in the outcome, and thus summarizes millions of predictors into several similarity matrices that are further used for prediction analysis. By using the similarity matrices, LMMs implicitly reduce the data dimension substantially and have natural advantages in modeling high-dimensional omics data. Despite their potentials, existing LMMs usually assumes that the effect sizes come from normal distributions that may not represent the true effect size distribution, and they usually overlooked the complex inter/intra-relationships among multi-omics data (e.g. methylation/genome interaction and genome/genome interaction) (Van Ijzendoorn *et al.* 2010, Misra *et al.* 2019). In addition, the vast amount of noise in multi-omics data can result in an inaccurate estimates of the similarities. These can all lead to reduced prediction performance.

To address these issues, we developed a two-step Bayesian Linear mixed model (TBLMM) for predictive modeling of multi-omics data. TBLMM first uses the Bayesian linear mixed model (BLMM)-based integrative framework to fuse multiple designated kernel functions, which can account for heterogeneous effects and interactions among multi-omics data, into one kernel for each genomic region. It then models the omics effects by directly utilizing the fused kernels from all genomic regions for prediction. In the following sections, we first provided the technical details of TBLMM framework, and then evaluated the predictive performance of the proposed method through extensive simulations. Finally, we demonstrated its practical utility by analyzing the multi-omics data obtained from the Alzheimer’s Disease Neuroimaging Initiative (ADNI) (Saykin *et al.* 2015).

## 2 Materials and methods

Similar to existing LMMs (Wang and Wen 2022b), we model the outcome as a sum of region-wise predictive effects: $Y=\sum_{m=1}^{M}F_{m}+\varepsilon_{n},\varepsilon_{n}∼N(0,I\sigma_{\varepsilon }^{2})$, where region can be defined based on biological annotations (e.g. genes and pathways) and $F_{m}$ is the joint predictive effect from all omics data for region *m*. Following the same idea from Hai and Wen (2020), we further decomposed the region-wise predictive effects $F_{m}$ into two parts, namely the large effects from a few predictors $X_{m}\beta_{m}$ and the small-to-moderate effects from a large number of predictors ($O_{m}$) that include the marginal effects from for each omics layer and their interactions:


(1) $$Y=\sum_{m=1}^{M}\left(X_{m}\beta_{m}+O_{m}\right)+\varepsilon_{n}=\sum_{m=1}^{M}\left(X_{m}\beta_{m}+\sum_{j\in S_{m}}o_{j}^{m}\right)+\varepsilon_{n},$$

where $X_{m}=[X_{ex}^{m},X_{me}^{m},\ldots ,X_{ge}^{m}]$ is the a $n\times p_{o}^{m}$ dimensional predictors with large effects from multi-omics data (e.g. gene expression, methylation, and genomics data) for region *m* and $\beta_{m}$ is their corresponding effect. $o_{j}^{m}∼N(0,K_{j}^{m}\sigma_{mj}^{2})$ and $S_{m}$ is the set of all omics effects considered (e.g. marginal predictive effects from genomic data and the interaction between genomic and methylation) in region *m*.

Whole-genome multi-omics data can be ultra-high dimensional with lots of noise regions (e.g. ∼20 000 genes for human genome), and thus directly estimating parameters in Equation (1) can be computationally demanding and the impact of noise is not negligible. Therefore, we proposed a two-step procedure, where the first step aims at reducing the number of parameters in Equation (1) while accounting for complex inter/intra-relationships among multi-omcis data and the second step focuses on prediction modeling. Specifically, for the first step in TBLMM, we propose to summarize the joint predictive effects from markers with small to moderate effects (i.e. $\sum_{j\in S_{m}}o_{j}^{m}$) through kernel fusion and eliminate potential markers with large effects (i.e. $X_{m}$) using a procedure that is similar to the C + T proposed by Privé *et al.* (2019). The second step utilizes the BLMM method that we proposed to model the outcome (Hai and Wen 2020), where fused kernel and the reduced set of markers with potential large effects are modeled.

### 2.1 Step 1: Multi-omics data integration for each region

In this step, we modeled the outcome using one region at a time, and our model for region *m* can be written as: $Y=X_{m}\beta_{m}+\sum_{j\in S_{m}}o_{j}^{m}+\varepsilon_{n}$. Biological systems are regularized at multiple levels that involve interactions between and within functional layers of omics data (Dahlin and Tantisira 2012). Suppose we consider $T_{1}^{m}$ different omics data (e.g. genomics, gene expression, and methylation data), $T_{2}^{m}$ within-layer and $T_{3}^{m}$ between layer interactions for region *m*. Our single-region model can be written as:


(2) $$Y=X_{m}\beta_{m}+\sum_{t=1}^{T_{1}^{m}}o_{t}^{m}+\sum_{t\prime =1}^{T_{2}^{m}}W_{t\prime }^{m}+\sum_{t\prime \prime =1}^{T_{3}^{m}}B_{t\prime \prime }^{m}+\varepsilon_{n},$$

where $o_{t}^{m}$ is the joint predictive effects from all markers in the *t*th omics layer for region *m*; $W_{t\prime }^{m}$ is the t′th within layer interactions for region *m*; and $B_{t\prime \prime }^{m}$ is the t″th between layer interactions for region *m*. Similar to BLMM proposed in Hai and Wen (2020), we used random effect terms to model these joint effects as $o_{t}^{m}∼N(0,K_{o,t}^{m}\sigma_{o,mt}^{2})$; $W_{t\prime }^{m}∼N(0,K_{w1,t\prime }^{m}\sigma_{w1,mt\prime }^{2}+K_{w2,t\prime }^{m}\sigma_{w2,mt\prime }^{2})$; and $B_{b,t\prime \prime }^{m}∼N(0,K_{b,t\prime \prime }^{m}\sigma_{b,mt\prime \prime }^{2})$. For the marginal predictive effects from each omics (i.e. $o_{t}^{m}$), we used a linear kernel function defined as $K_{o,t}^{m}(Z_{mt})={\left(\frac{1}{\sqrt{p_{mt}}}Z_{mt}^{k}\right)}^{T}(\frac{1}{\sqrt{p_{mt}}}Z_{mt}^{l})$, where *p_mt_* is the number of variants for *t*th omics layer; $Z_{mt}^{k}$ and $Z_{mt}^{l}$ are the vectors of *t*th omics data for individual *k* and *l*. For the within layer interactions, we considered two kernels, including the polynomial with 2 degrees of freedom and the saturating pathway kernel. The polynomial with 2 degrees of freedom that can capture the pairwise interactions among predictors (Henderson 1985, Su *et al.* 2012, Bloom *et al.* 2015, Weissbrod *et al.* 2016) is defined as $K_{w1,t\prime }^{m}(Z_{mt\prime })={\left({\left(\frac{1}{\sqrt{p_{mt\prime }}}Z_{mt\prime }^{k}\right)}^{T}(\frac{1}{\sqrt{p_{mt\prime }}}Z_{mt\prime }^{l})\right)}^{2}$. The saturating pathway kernel (Williams and Rasmussen 2006), also known as the neural network kernel, can capture pairwise multiplicative interactions and is defined as $K_{w2,t\prime }^{m}(Z_{mt\prime };\theta_{mt\prime }^{w2w})=\frac{1}{2\pi }\cdot {\;\text{sin}\;}^{-1}(\frac{\frac{1}{p_{mt\prime }}{\left(Z_{mt\prime }^{k}\right)}^{T}Z_{mt\prime }^{l}}{\sqrt{\left(\theta_{mt\prime }^{w2w}+|Z_{mt\prime }^{k}{|}^{2}/p_{mt\prime })(\theta_{mt\prime }^{w2w}+|Z_{mt\prime }^{l}{|}^{2}/p_{mt\prime }\right)}})$, where $\theta_{mt\prime }^{w2w}$ is the bandwidth parameter for omics layer t′ on region *m*, which specifies the rate of decay of the covariance. For our method, we set this parameter to be 1. The kernel used to capture between layer interactions is defined as $K_{b,t\prime \prime }^{m}=K_{t\prime \prime {\;}_{1}}^{m}{}^{\circ}K_{t{\prime \prime }_{2}}^{m}$, where ° denotes the Hadamard product operation; and $K_{t\prime \prime {\;}_{1}}^{m}$$K_{t\prime \prime {\;}_{2}}^{m}$ are covariance matrices for omics layers $t\prime \prime_{1}$ and $t\prime \prime_{2}$.

Based on Equation (2), the variance–covariance matrix of the outcome can be viewed as a sum of omics-specific and interaction kernels $\sum^{m}=\sum_{t=1}^{T_{1}^{m}}\sigma_{o,mt}^{2}K_{o,t}^{m}+\sum_{t\prime =1}^{T_{2}^{m}}\sigma_{w1,mt\prime }^{2}K_{w1,t\prime }^{m}+\sum_{t\prime =1}^{T_{2}^{m}}\sigma_{w2,mt\prime }^{2}K_{w2,t\prime }^{m}+\sum_{t\prime \prime =1}^{T_{3}^{m}}\sigma_{b,mt\prime \prime }^{2}K_{b,t\prime \prime }^{m}+I_{n}\sigma_{0}^{2}$, where $\sigma_{ml}^{2}\in [\sigma_{o,m1}^{2},\ldots ,\sigma_{w1,m1}^{2},\ldots ,\sigma_{w2,m1}^{2},\ldots ,\sigma_{b,m1}^{2}],\;l\in \{1,\ldots ,L=T_{1}^{m}+T_{2}^{m}+T_{3}^{m}\}$ is the magnitude of omics effects in region *m*, known as $\sigma_{g}^{2}$ in the genomic Best Linear Unbiased Prediction (gBLUP) models. All coefficients ($\sigma_{ml}^{2}$) are constrained to be non-negative. As not all of the considered effects from region *m* are predictive, following the same procedure in Hai and Wen (2020), we adopted the spike and slab prior for $\sigma_{ml}^{2}$ and inferred them using the computationally efficient variational Bayes algorithm [detailed derivation can be referred to Hai and Wen (2020)]. Let $K_{l}^{m}\in [K_{1}^{m},\ldots ,K_{w1,1}^{m},\ldots ,K_{w2,1}^{m},\ldots ,K_{b,1}^{m},\ldots ],l=1,\ldots ,L$ denote the set of candidate kernels derived from *L* different omics data sources and corresponding interactions. The final fused kernel for region *m* is defined as a linear combination of multiple candidate kernels $K^{m}=\sum_{l=1}^{L}w_{l}^{m}K_{l}^{m}$, where $w_{l}^{m}=\frac{\sigma_{ml}^{2}}{\sum_{l=1}^{L}\sigma_{ml}^{2}}$ and $\sum_{l=1}^{L}w_{l}^{m}=1$. The fused kernel $K^{m}$ for region *m* is further used for prediction, and thus Equation (1) is equivalent to


(3) $$Y=\sum_{m=1}^{M}\left(X_{m}\beta_{m}+O_{m}\right)+\varepsilon_{n},\text{with}\;O_{m}∼N(0,K^{m}\sigma_{m}^{2}).$$

To accommodate various disease models, TBLMM allows all markers to have both large and small effects. However, it is quite likely that only a small proportion of them have large effects in practice. Including all markers as if they had large effects in the TBLMM model (i.e. $X_{m}\beta_{m}$) requires dramatically more computational resources, especially when working with high-dimensional multi-omics data. As discussed in Hai and Wen (2020), it is likely that markers with large effects can be detected from simply marginal association analysis. Therefore, we selected a small fraction of predictors for each region by using a procedure that is similar to the C + T method in Privé *et al.* (2019) to reduce computational burden of TBLMM. Specifically, we used a univariate analysis (e.g. linear regression) to estimate effect size of each predictor in region *m*. A fraction of predictors (e.g. 5%) with the largest effects within each region are selected, and the $n\times p_{o}^{m}$ dimensional *X_m_* matrix in Equation (1) is reduced to $X_{m}^{r}$ that has a $n\times p_{r}^{m}$ dimension (e.g. $m_{r}=0.05m_{o}$).

### 2.2 Step 2: Risk prediction with multi-omics data

With fused kernel and selected predictors for each region, Equation (1) can be written as


(4) $$Y=\sum_{m=1}^{M}\left(X_{m}^{r}\beta_{m}^{r}+O_{m}\right)+\varepsilon_{n},$$

where $O_{m}∼N(0,K^{m}\sigma_{m}^{2})$; $K^{m}$ is the fused kernel matrix for region *m*, and $X_{m}^{r}$ is the selected predictors for region *m*. Similar to Hai and Wen (2020), we used the Bernoulli-Gaussian prior and spike and slab prior for each $\beta_{m}^{r}$ and $\sigma_{m}^{2}$, respectively. All the parameters are inferred using the variational Bayes algorithm. Specifically, we re-parameterized Equation (4) as $Y=\sum_{m=1}^{M}\left(X_{m}^{r}\Gamma_{m}\beta_{m}^{r}+O_{m}\right)+\varepsilon_{n}$, where $\Gamma_{m}=$ diag($\gamma_{m}$) and $\gamma_{m}={\left(\gamma_{m1},\gamma_{m2},\ldots ,\gamma_{mp_{m}}\right)}^{T}$ is a vector of binary variables indicating whether each predictor is selected. To select predictive markers, Bernoulli–Gaussian priors are set for $\beta_{m}^{r}$ and *γ_m_* (i.e. $\beta_{m}^{r}∼N(0,I_{r}\sigma_{\beta_{m}}^{2})$ and $\gamma_{mj}∼\text{Bernoulli}(\delta_{0})$). To select predictive regions (i.e. $O_{m}$), a spike and slab prior is used, where $O_{m}|K^{m},\sigma_{m}^{2}∼℘(r_{m})N(0,K^{m}\sigma_{m}^{2})+(1-℘(r_{m}))\delta_{1},\;r_{m}∼\mathrm{Bernoulli}(\delta_{0}),\;℘(r_{m})$ is the proportion, and $\delta_{1}=0$. This allows TBLMM to further select predictive regions and predictors when multiple regions are considered simultaneously.

## 3 Simulation study

We conducted extensive simulation studies to evaluate the performance of TBLMM under various disease models and noise levels. We further compared TBLMM with OmicKrig (Wheeler *et al.* 2014), a widely used method for risk prediction on multi-omics data. In addition, to evaluate the impact of multi-omics integration on prediction, we analyzed each layer of omics data separately using TBLMM. Furthermore, to gauge the impacts of including non-linear kernels, we also analyzed the simulated data using TBLMM with only linear kernel (TBLMM-LIN). Three types of omics data including gene expression, methylation and genomic data were considered as inputs for the following simulation studies. To mimic the real human genome and gene expression levels, we selected genomics and gene expression data from ADNI (*n* = 712). The number of genetic variants per gene ranges from around 200 to 16 000 with an average of around 1300. For methylation data, we generated it using the methylKit R package (Akalin *et al.* 2012). For TBLMM, we considered each gene as a region, and treated the gene expression levels as additive effects. For all the simulations described below, two genes were randomly chosen to be causal. We randomly selected 80% of samples to train the model and used the remaining to calculate the Pearson correlation and root mean square errors (RMSEs). We repeated each simulation scenario 100 times.

### 3.1 Scenario 1: the impact of disease model

In the first set of simulations, we evaluated the performance of TBLMM under different disease models, including (i) only single omics data contributes to disease risk and (ii) multiple types of omics data jointly contribute to disease risk. Genetic, gene expression, and methylation data from 30 randomly selected genes were extracted and two of these genes were set as causal.

#### 3.1.1 The outcome is affected by single-layer omics data

We first evaluated the performance of TBLMM when only single-layer omic data contributes to disease risk. We considered both linear and non-linear predictive effects and simulated the outcomes as


(5) $$Y=\sum_{m=1}^{2}I^{G}X_{m}^{G}\beta_{m}^{G}+\sum_{m=1}^{2}I^{M}X_{m}^{M}\beta_{m}^{M}+\sum_{m=1}^{2}I^{E}X_{m}^{E}\beta_{m}^{E}+\sum_{m=1}^{2}o_{m}^{G}+\sum_{m=1}^{2}o_{m}^{M}+\varepsilon ,$$

where $\varepsilon ∼N(0,\sigma_{\varepsilon }^{2})$. We used superscript to denote the relevant omics data, where *G*, *M*, *E* respectively denote genomics, methylation, and gene expression data. $I_{m}^{j},j\in \{G,M,E\}$ is an indicator function and it is equal to 1 if the relevant omics on region *m* contributes to the outcome. $X_{m}^{j},j\in \{G,M,E\}$ denotes the predictors from one of the omics data on region *m* and $\beta_{m}^{j}$ represents its corresponding effect. $o_{m}^{j}∼N(0,K_{m}^{j}\sigma_{m}^{2,j}),j\in \{G,M\}$ denotes the joint predictive effects from all predictors with small-to-moderate effects in region *m*. We considered five types of disease models, including *S*_1_: only genomics data contributes to the outcome in an additive manner ($I_{m}^{G}\neq 0,\;\sigma_{m}^{2,G}\neq 0$, and $K_{m}^{G}$ is calculated using a linear kernel); *S*_2_: only transcriptomics data contributes to the outcome ($I_{m}^{E}\neq 0$); *S*_3_: only methylation data contributes to the outcome in an additive manner ($I_{m}^{M}\neq 0,\;\sigma_{m}^{2,M}\neq 0$ and $K_{m}^{M}$ is calculated using a linear kernel); *S*_4_: only pairwise interaction within genomics data contributes to the outcome ($\sigma_{m}^{2,G}\neq 0$ and $K_{m}^{G}$ is calculated using a polynomial kernel with 2 degrees of freedom); and *S*_5_ only non-linear effects within genomics data contributes to the outcome ($\sigma_{m}^{2,G}\neq 0$ and $K_{m}^{G}$ is calculated using a neural network kernel). We set the total heritability ranging from 0.2 to 0.6, and the details are summarized in Supplementary Table S1.

**Figure 1.**
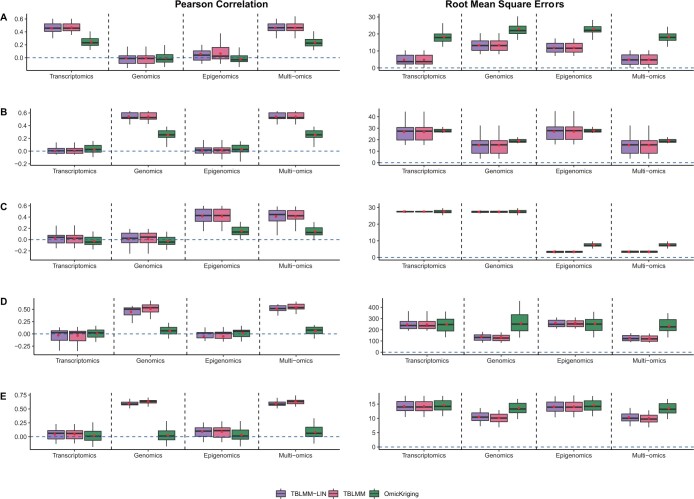
The performance of TBLMM when the outcome is affected by single omics ($h^{2}=0.4$). Five settings were considered, including the outcomes were simulated with only gene expression data (A), only genomics data with linear effects (B), only methylation data with linear effects (C), pairwise interaction effects within genomic data (D), and non-linear predictive effects from only genomics data (E). *Transcriptomics*: using only trasncriptomic data for prediction; *Genomics*: using only genomic data for prediction; *Epigenomics*: using only epigenomic data for prediction; and *Multi-omics*: using all three layers of omics data for prediction.

The Pearson correlations and RMSEs for heritability of 0.2, 0.4, and 0.6 are shown in Supplementary Fig. S1, Fig. 1, and Supplementary Fig. S2, respectively. When single omics data contributes to disease risk in an additive manner (Fig. 1A–C and Supplementary Figs S1 and S2), TBLMM performs similarly to TBLMM-Lin, suggesting that TBLMM can select the most appropriate kernels in a data-driven manner. In addition, we noticed that TBLMM also has similar performance to the models where only relevant omics is included, which indicates that TBLMM can tease out the impact of noise and exclude non-relevant omics automatically when building prediction models. Comparing to the OmicKrig method, TBLMM consistent outperforms it, which is likely due to the fact that TBLMM allows for variable selection and select the appropriate kernels in a data-driven manner. When only non-linear predictive effects are present (Fig. 1D and E and Supplementary Figs S1 and S2), TBLMM tends to perform better than the existing method and TBLMM-LIN. This is mainly because TBLMM is designed to capture interaction effects through kernelizing the variance–covariance matrix by using polynomial and neural network kernels. In practice, the underlying mechanisms of diseases are usually unknown in advance. Therefore, it is important that TBLMM can have robust and accurate prediction performance regardless of the types of predictive effects.

#### 3.1.2 The outcome is affected by multiple omics data

We evaluated the performance of TBLMM when multiple types of omics data contribute to the outcomes independently or jointly. We added the interaction between omics ($I_{m}^{O}o_{m}^{O}$) into Equation (5), and simulated the outcomes as $Y=\sum_{m=1}^{2}I^{G}X_{m}^{G}\beta_{m}^{G}+\sum_{m=1}^{2}I^{M}X_{m}^{M}\beta_{m}^{M}+\sum_{m=1}^{2}I^{E}X_{m}^{E}\beta_{m}^{E}+\sum_{m=1}^{2}o_{m}^{G}+\sum_{m=1}^{2}o_{m}^{M}+\sum_{m=1}^{2}I_{m}^{O}o_{m}^{O}+\varepsilon$. $o_{m}^{O}$ is the interaction effects between the genotype and methylation for region *m* and $o_{m}^{O}∼N(0,K_{m}^{O}\sigma_{m}^{2})$. $K_{m}^{O}=K_{m}^{G}{}^{\circ}K_{m}^{M}$ is the epistatic effect for region *m*, where $K_{m}^{G}=X_{m}^{G}{\left(X_{m}^{G}\right)}^{T}/p_{m}^{G}$ and $K_{m}^{M}=X_{m}^{M}{\left(X_{m}^{M}\right)}^{T}/p_{m}^{M}$. $I_{m}^{O}$ is an indicator function and it is 1 if the between omics interaction is present. In this simulation, we mainly considered two situations, including *S*_6_: multiple types of omics data contribute to the outcome independently ($I_{m}^{O}=0$); and *S*_7_: multiple types of omics data contribute to the outcome jointly with interactions between different layers of omics data present ($I_{m}^{O}\neq 0$). Similar as the above, we ranged the heritability from 0.2 to 0.6, and the details are in Supplementary Table S1.

**Figure 2.**
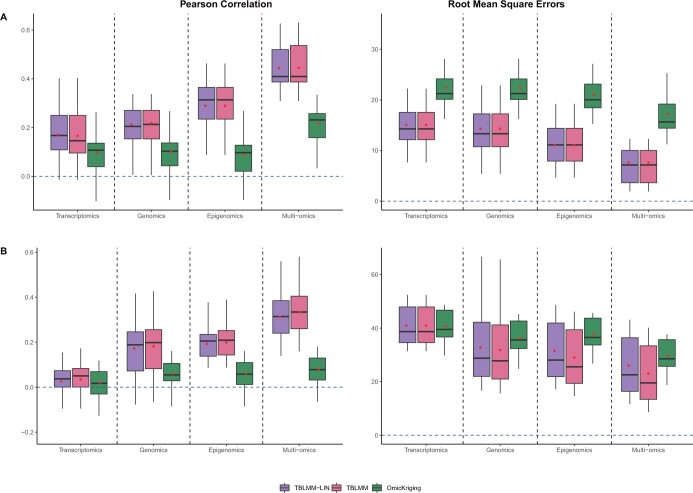
The performance of TBLMM when the outcome is affected by multiple omics ($h^{2}=0.4$). Two settings were considered, including multiple omics data contribute to the outcomes independently (A) and only interactions between genomics and methylation contribute to the outcome (B). *Transcriptomics*: using only trasncriptomic data for prediction; *Genomics*: using only genomic data for prediction; *Epigenomics*: using only epigenomic data for prediction; and *Multi-omics*: using all three layers of omics data for prediction.

The Pearson correlations and RMSEs for heritability of 0.2, 0.4, and 0.6 are shown in Supplementary Fig. S3, Fig. 2, and Supplementary Fig. S4, respectively. When multiple layers of omics data independently contribute to the outcome, TBLMM performs similarly to TBLMM-Lin as expected, but it performs substantially better than the models that only uses single-layer omics data. This suggests that jointly analyzing all omics data can benefit prediction analyses when more than one layer of omics data contribute to the outcome. When multiple layers of omics data affect disease risk through interactions (i.e. Fig. 2B and Supplementary Figs S3 and S4), TBLMM outperforms the other methods, including TBLMM-Lin. TBLMM-Lin assumes that each layer of omics data contributes independently to the outcome and thus fails to capture the interaction effects between omic layers, whereas TBLMM are designed to capture the interactions through non-additive kernel functions and it has the capacity in selecting the most appropriate kernels for prediction. Regarding the comparison between TBLMM and OmicKrig, TBLMM significant outperforms OmicKrig, which not only due to the fact that TBLMM can select the appropriate kernels to model complex types of predictive effects but also because TBLMM can tease out the impact of noise.

### 3.2 Scenario 2: the impact of noise regions

In this set of simulations, we evaluated the impact of noise genes on the performance of TBLMM by gradually increasing the number of noise genes from 28 to 48 (i.e. the total number of genes changes from 30 to 50). We simulated outcomes under the setting of *S*_6_ in Supplementary Table S1, where all omics contributed to the outcome independently. Two genes were randomly selected as the causal, and the heritability ranged from 0.2 to 0.6.

**Figure 3.**
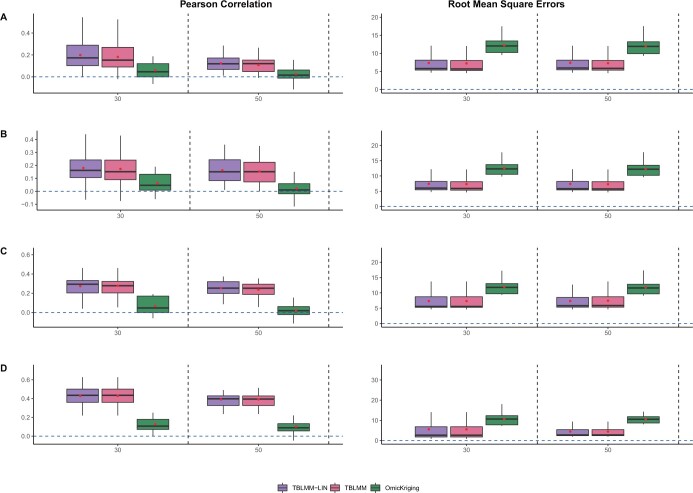
The effects of the number of noise regions ($h^{2}=0.4$). The outcomes were simulated with all omics are predictive, and they were modelled with only gene expression data (A), only genomics data (B), only methylation data (C), and all omics (D).

Pearson correlations and RMSEs for heritability of 0.2, 0.4, and 0.6 are shown in Supplementary Fig. S5, Fig. 3, and Supplementary Fig. S6, respectively. Similar to the results shown in Section 3.1.2, when different layers of omics data contributed to the outcome independently, TBLMM and TBLMM-Lin perform similarly and their accuracy is much higher than those models built with single layers of omics data (Fig. 3D versus Fig. 3A–C). We noticed that as the amount of noise increases, the performance of TBLMM remains relatively stable, indicating that TBLMM has robust performance against noise, which is likely due to its variable selection technique implemented. Indeed, the average sensitivity and specificity for TBLMM are 68% and 72%, respectively. Although the sensitivity and specificity of the selection can be impacted by the number of noise genes and the effect sizes, TBLMM in general have the capacity to correctly select predictive genes and filter out the noise. We considered this property important, as multi-omics data tend to have lots of noise in practice.

## 4 Real data application

To demonstrate the practical utility of TBLMM, we predicted the positron emission tomography (PET) imaging outcomes using the whole-genome sequencing and gene expression data obtained from the ADNI study. ANDI is a multi-site longitudinal study for the prevention and treatment of Alzheimer’s Disease (AD) (Mueller *et al.* 2005). Whole genome sequencing (WGS) of 818 ADNI-1/GO/2 samples was performed on blood-derived DNA samples, where the Illumina HiSeq2000 was used. Gene expression profiling was performed on 811 ADNI participants included in the WGS study. The Affymetrix Human Genome U219 Array was used for expression profiling (Saykin *et al.* 2015). After Quality Control filtering, a total of 712 samples with both genomic data and gene expression were used in this study.

For this analysis, we focused on the baseline data, and the sample sizes for FDG and AV45 are 639 and 501, respectively. The distributions of AV45 and FDG are shown in Supplementary Fig. S7. We used 57 AD susceptibility genes selected based on the existing literature, and their details are shown in Supplementary Table S2. To avoid over-fitting, 80% of the data was used to train the model for AV45 and FDG, and Pearson correlations and RMSEs were calculated based on the remaining 20% of the data. To avoid the chance findings, this process was replicated 100 times. For comparison purposes, we also built prediction models using the OmicKriging method.

**Figure 4.**
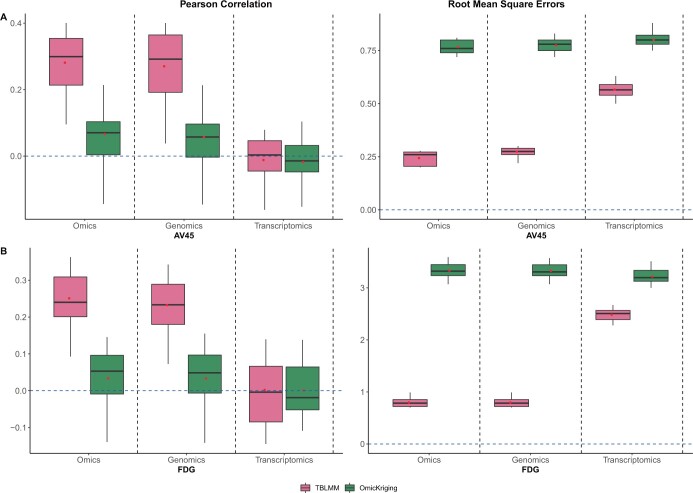
Comparison of prediction performance of proposed method with existing methods for PET imaging outcomes: (A) AV45 and (B) FDG. *Transcriptomics*: using only trasncriptomic data for prediction. *Genomics*: using only genomic data for prediction. *Omics*: using both genomics and transcriptomics data for prediction.

The prediction accuracies are shown in Fig. 4. TBLMM has achieved higher prediction accuracy than that of the OmicKriging method for both AV45 and FDG, indicating that the prediction can be benefited from excluding noise. Comparing the models built with omics data and the ones built with single-layer omics data, we have found that the model built with genomic data only and TBLMM have achieved comparable performance, indicating that the variance of outcomes can mainly be explained by genetic effects. To further explore our prediction models, we calculated the probability of each gene being selected by TBLMM (Supplementary Table S2). *APOC* and *APOE* have been the most frequently selected genes for both AV45 and FDG (selection probability larger than 85%), and more than 75% of the genes have been selected less than 50% times.

## 5 Discussions

In this article, we have proposed a novel TBLMM model to predict the outcomes of interest using high-dimensional multi-omics data. Our TBLMM is a two-step procedure, where the first step focuses on integrating information from multi-omics data via kernel fusion and the second step aims at using a non-parametric Bayesian linear mixed model to detect predictors and model their predictive effects that can be of various forms. Our proposed TBLMM is flexible and can accommodate various disease models. For example, through both sparsity regression and the random effect terms, it can easily be used for diseases with omnigenic architecture. By simultaneously considering multiple kernels and using a data-driven way to select the most appropriate kernel, TBLMM can not only select relevant predictors but also capture both complex additive and non-additive omics effects simultaneously. Through extensive simulations, we have showed that TBLMM outperforms OmicKrig, and it also has better and robust performance than those methods where only additive effects are considered or only single omics is used for prediction. Our real data analysis also reveals that TBLMM has better prediction accuracy than its competing methods.

Multi-omics data allows for the investigation of the flow of information from one omics level to the other (e.g. genome to proteome) and can help narrowing the gap between genotype and phenotype (Wang *et al.* 2015, Das *et al.* 2020). Our framework includes the data integration step to consider omics effects from different omics layers. It is built within the BLMM framework, where predictors from different omics layers are allowed to have both large effects that are modeled by the fixed effect term (Xβ) and small-to-moderate effects that are modeled by random effect terms ($o∼N(0,K\sigma^{2})$). Mounting evidences suggest that interactions widely exist and they can provide additional information for enhancing the performance of risk prediction (González-Reymúndez *et al.* 2017, Hawe *et al.* 2019, Li *et al.* 2020). For example, Fisher *et al.* (2018) found that methylation-genotype interaction plays an important role in changing levels of triglyceride. To explicitly model these interaction effects, during omics integration, TBLMM further uses random effect terms to capture both within and between omics interactions, where the variance-covariance is modeled using three non-linear kernels, including polynomial kernel with 2 degrees of freedom, the neural network kernel, and the Hadamard product between linear kernels. With spike and slab and Bernoulli–Gaussian priors imposed, the TBLMM can select relevant omics (e.g. genotypes or methylation) and their corresponding types of predictive effects (e.g. linear or pairwise interaction), which can provide valuable information for the understanding of complex biological processes. Our simulation studies have shown that TBLMM significantly outperforms those single-omics-based methods and it has also better performance than those where only additive effects are considered, indicating integrating multi-omics data is important for improved prediction.

Variable selection and dimensional reduction are considered as an indispensable step for the analysis of multi-omics data, and they have been successfully used to improve prediction accuracy (Liu *et al.* 2017, Duan *et al.* 2018, Wang and Wen 2022a). The TBLMM framework for multi-omics data modeling is developed based on the BLMM (Hai and Wen 2020), which uses “Bernoulli-Gaussian prior” and “spike and slab prior” to facilitate variable selections. Compared to existing dimension reduction methods (e.g. CCA) that treat variable selection and prediction as separate jobs, the TBLMM method streamlines this process where the variables are selected to maximize the prediction accuracy. It can detect predictors with relatively large effects and also find regions with significant contributions. It has an average of 70% sensitivity and specificity. With noise regions/variants being excluded, the TBLMM has yielded a much higher prediction accuracy as compared to OmicKrig. In addition, with predictive variables detected, TBLMM provides us with a better interpretation of the prediction models and can shed light on the underlying mechanisms. For example, in the prediction analysis of PET imaging outcomes, we found that genotype of the two-well known AD-related genes (i.e. *APOE* and *APOC*) are consistently selected as important predictors. The variable selection technique implemented in TBLMM can not only improve the prediction accuracy but also facilitate the interpretation of the model, making it practically useful.

TBLMM has several attractive features to reduce computational cost for modeling the high-dimensional multi-omics data. First, similar to LMM extensions (Speed and Balding 2014, Li *et al.* 2020), TBLMM uses random effect terms to model the cumulative predictive effects of all the markers within each genetic region, and thus reduces the data dimension substantially. Second, it adopts a two-step procedure to summarize the joint effects in the region first to further reduce the number of parameters in Equation (2). Indeed, directly solving Equation (2) is extremely computationally demanding. To illustrate, suppose we have three layers of omics data and consider their independent predictive effects and within/between omics interactions. We would have 12 (i.e. 3 for independent predictive effects, 6 for within layer, and 3 for between layer interactions) random effect terms for each region. Given *M* regions, a total of 12*M* random effects need to be estimated. To alleviate the computational burden, in the first step of TBLMM, we used a similar idea in multi-kernel learning and fused the 12 kernels into one for each region by using the BLMM model. Therefore, the final prediction model in Step 2 would only have *M* random effects to estimate. Third, the TBLMM model allows markers to have large predictive effects and modeled them using the fixed effects (i.e. Xβ). While this provides modeling flexibility for outcomes with various underlying mechanisms, it introduces a substantial computational burden due to the ultra-high dimension of multi-omics data. To enable efficient computation, we used a similar idea used in C + T (Privé *et al.* 2019) to select a small fraction of markers with potential large effects and estimated parameters using variational Bayesian algorithm rather than the traditional Markov chain Monte Carlo. This allows TBLMM to not only capture the predictive effects from these markers but also make it computationally feasible. The computational time as the number of predictors increases is shown in Supplementary Fig. S8.

While TBLMM is efficient and accurate in prediction modeling of multi-omics data, there are several limitations. First, TBLMM is primarily designed for continuous traits as it is built based on non-parametric Bayesian linear mixed model. It would be interesting to further extend TBLMM for categorical outcomes, where Bayesian generalized linear models are considered. Second, TBLMM only focuses on the between layer interactions within each region, and it is natural to further extend it to consider interactions among different regions. Nevertheless, TBLMM presents a valuable framework for the analysis of multi-omics data. It can jointly model both linear and non-linear effects from multi-omics data, and achieve higher and robust prediction accuracy as compared to competing methods.

## Supplementary Material

btad647_Supplementary_Data
