# Supplementary material for: Bayesian linear mixed model with multiple random effects for prediction analysis on high-dimensional multi-omics data
Source: Bioinformatics. 2023 Oct 26;39(11):btad647. doi: 10.1093/bioinformatics/btad647 (PMC10627352; doi:10.1093/bioinformatics/btad647)
Supplement: btad647_Supplementary_Data [file btad647_supplementary_data.pdf]

Bayesian linear mixed model with multiple random effects for prediction analysis on high-dimensional multi-omics data

Yang Hai, Jixiang Ma, Kaixin Yang, Yalu Wen*

A Supplementary Tables

Table S1. Simulation settings

Model	Scenario	Genomics (h)	Transcriptomics (h)	Epigenomics (h)	Interaction (h)
S_1	1	0.2	0	0	0
S_1	2	0.4	0	0	0
S_1	3	0.6	0	0	0
S_2	1	0	0.2	0	0
S_2	2	0	0.4	0	0
S_2	3	0	0.6	0	0
S_3	1	0	0	0.2	0
S_3	2	0	0	0.4	0
S_3	3	0	0	0.6	0
S_4	1	0	0	0	0.2
S_4	2	0	0	0	0.4
S_4	3	0	0	0	0.6
S_5	1	0	0	0	0.2
S_5	2	0	0	0	0.4
S_5	3	0	0	0	0.6
S_6	1	0.15	0.15	0.15	0
S_6	2	0.2	0.2	0.2	0
S_6	3	0.25	0.25	0.25	0
S_7	1	0	0	0	0.2
S_7	2	0	0	0	0.4
S_7	3	0	0	0	0.6

Note: There are seven simulation models: S_1: only genomics data contributes to the outcome in an additive manner; S_2: only transcriptomics data contributes to the outcome; S_3 : only methylation data contributes to the outcome in an additive manner; S_4: only pairwise interaction within genomics data contributes to the outcome; S_5: only non-linear effects within genomics data contributes to the outcome; S_6: multiple types of omics data contribute to the outcome independently; and S_7: multiple types of omics data contribute to the outcome jointly with interactions between different layers of omics data present.

Table S2. The summary of selection for genes used in real data analysis

Chr	Gene	Genomics	Transcriptomics	Additive	Random
1	COL11A1	Included	Included	0.10	0.40
1	FCER1G	Included	N/A	0.05	0.60
1	GBP2	Included	Included	0.05	0.60
1	HSD11B1	Included	Included	0.10	0.45
1	PARP1	Included	Included	0.05	0.15
1	POU2F1	Included	Included	0.05	0.30
1	NGF	Included	N/A	0.05	0.30
2	LHCGR	Included	N/A	0.05	0.45
2	LRP2	Included	Included	0.05	0.40
3	APOD	Included	Included	0.40	0.50
3	SST	Included	N/A	0.05	0.05
4	ALB	Included	Included	0.55	0.65
4	COL25A1	Included	Included	0.05	0.45
5	ADRB2	Included	N/A	0.05	0.50
5	ARSB	Included	Included	0.05	0.60
5	FGF1	Included	Included	0.50	0.70
5	FGF10	Included	N/A	0.00	0.00
5	FGF10-AS1	Included	N/A	0.00	0.00
5	FGF18	Included	N/A	0.00	0.00
5	NDUFS4	Included	N/A	0.05	0.40
5	PPP2R2B-IT1	Included	N/A	0.00	0.00
6	HSPA1A	Included	Included	0.05	0.35
6	MICA	Included	Included	0.05	0.55
6	MICAL1	Included	N/A	0.00	0.00
6	NBAT1	Included	N/A	0.00	0.00
6	TBP	Included	Included	0.20	0.20
6	TBPL1	Included	N/A	0.00	0.00
6	TREM2	Included	Included	0.05	0.20
7	CAV1	Included	Included	0.10	0.40
7	PON3	Included	Included	0.05	0.25
7	RELN	Included	Included	0.05	0.05
8	ADAM9	Included	Included	0.35	0.55
8	NAT1	Included	N/A	0.05	0.20
8	NRG1	Included	Included	0.05	0.15
9	DFNB31	Included	N/A	0.15	0.50
9	HSPA5	Included	Included	0.10	0.30
9	POMT1	Included	Included	0.05	0.35
9	RXRA	Included	Included	0.40	0.35
9	TLR4	Included	Included	0.05	0.15
10	CACNB2	Included	Included	0.30	0.65
10	MINPP1	Included	N/A	0.15	0.60
10	TET1	Included	Included	0.05	0.20
11	APOC3	Included	N/A	0.00	0.00
11	HBG2	Included	Included	0.05	0.55
12	ATF7	Included	Included	0.25	0.65
12	ATF7IP	Included	Included	0.00	0.00
12	SLC11A2	Included	Included	0.05	0.10
13	KLF5	Included	Included	0.05	0.60
14	HNRNPC	Included	Included	0.00	0.00
14	MTHFD1	Included	N/A	0.05	0.45
14	PNP	Included	N/A	0.00	0.00
19	APOC1	Included	Included	0.85	0.95
19	APOE	Included	N/A	0.90	0.85
19	APOC1P1	Included	N/A	0.00	0.00
19	APOC2	Included	Included	0.00	0.00
19	APOC4	Included	N/A	0.00	0.00
19	TOMM40	Included	N/A	0.50	0.60

Note: N/A: not available; **Additive**: $\boldsymbol{X\beta}$ part of TBLMM; **Random**: $\boldsymbol{O}_m$ part of TBLMM.

B Supplementary Figures

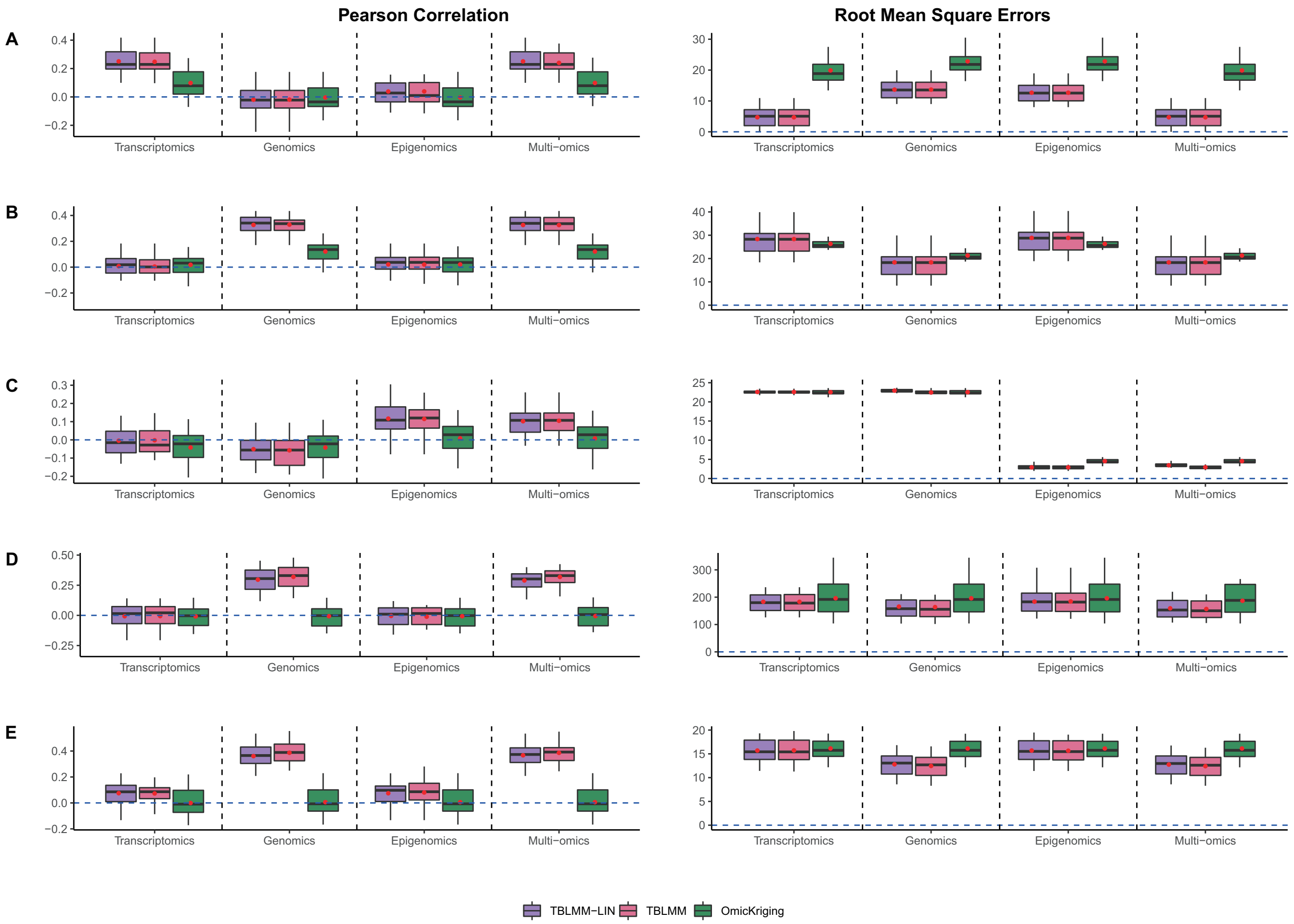

Fig. S1. The performance of TBLMM when the outcome is affected by single omics ($h^2 = 0.2$). Five settings were considered, including the outcomes were simulated with only gene expression data (**A**), only genomics data with linear effects (**B**), only methylation data with linear effects (**C**), pairwise interaction effects within genomic data (**D**), and non-linear predictive effects from only genomics data (**E**).

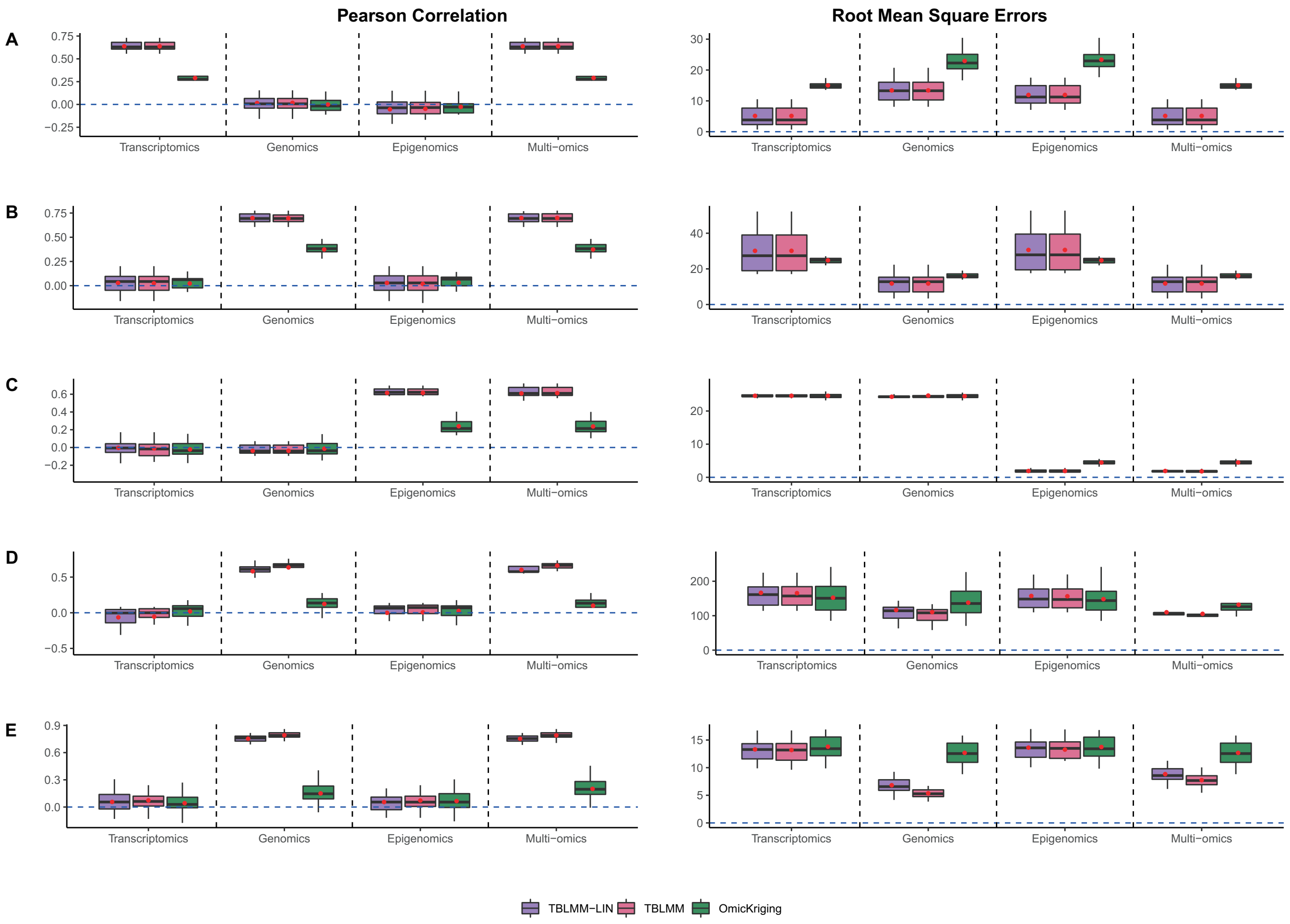

Fig. S2. The performance of TBLMM when the outcome is affected by single omics ($h^2 = 0.6$). Five settings were considered, including the outcomes were simulated with only gene expression data (**A**), only genomics data with linear effects (**B**), only methylation data with linear effects (**C**), pairwise interaction effects within genomic data (**D**), and non-linear predictive effects from only genomics data (**E**).

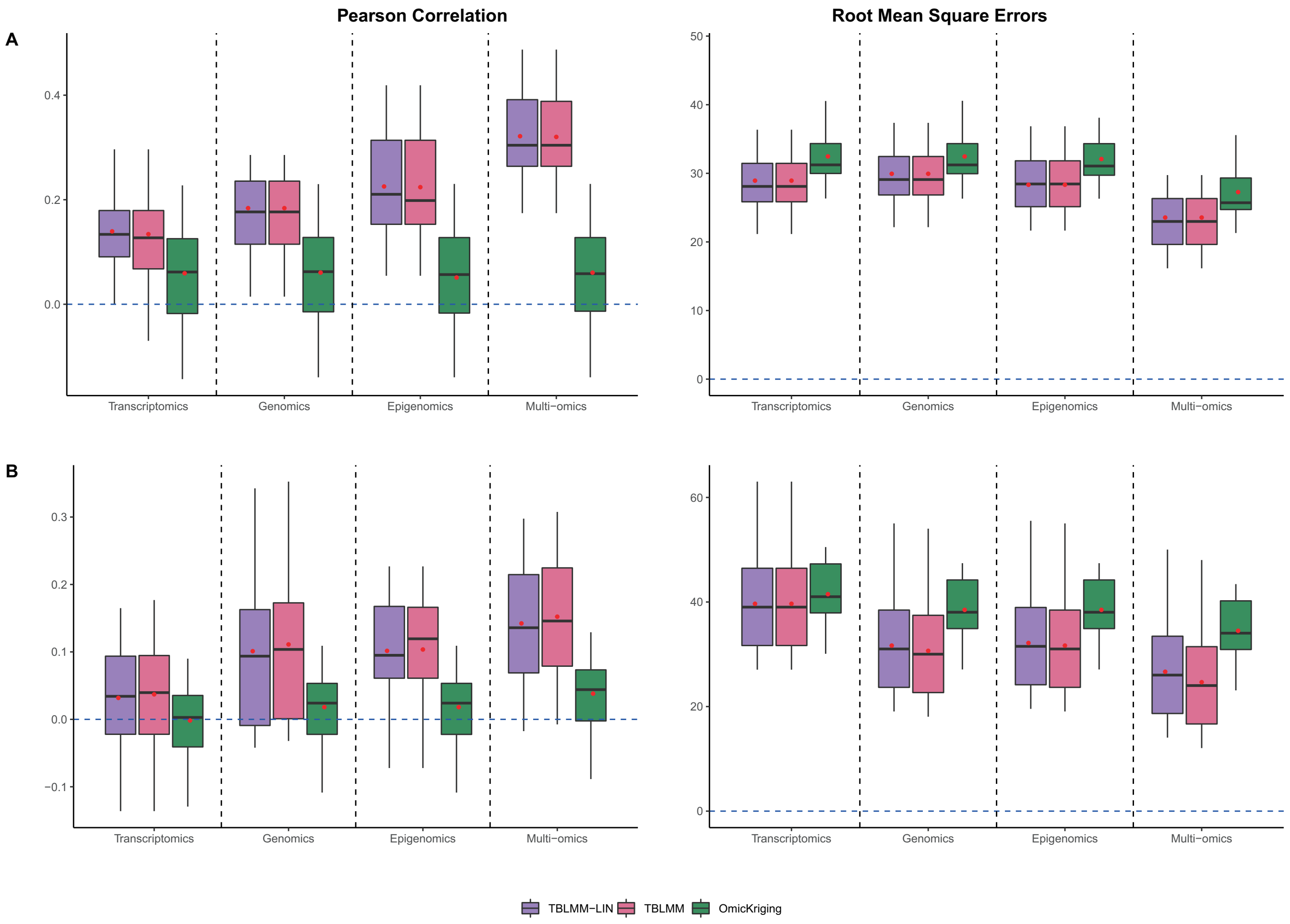

Fig. S3. The performance of TBLMM when the outcome is affected by multiple omics ($h^2 = 0.2$). Two settings were considered, including multiple omics data contribute to the outcomes independently (**A**) and only interactions between genomics and methylation contribute to the outcome (**B**).

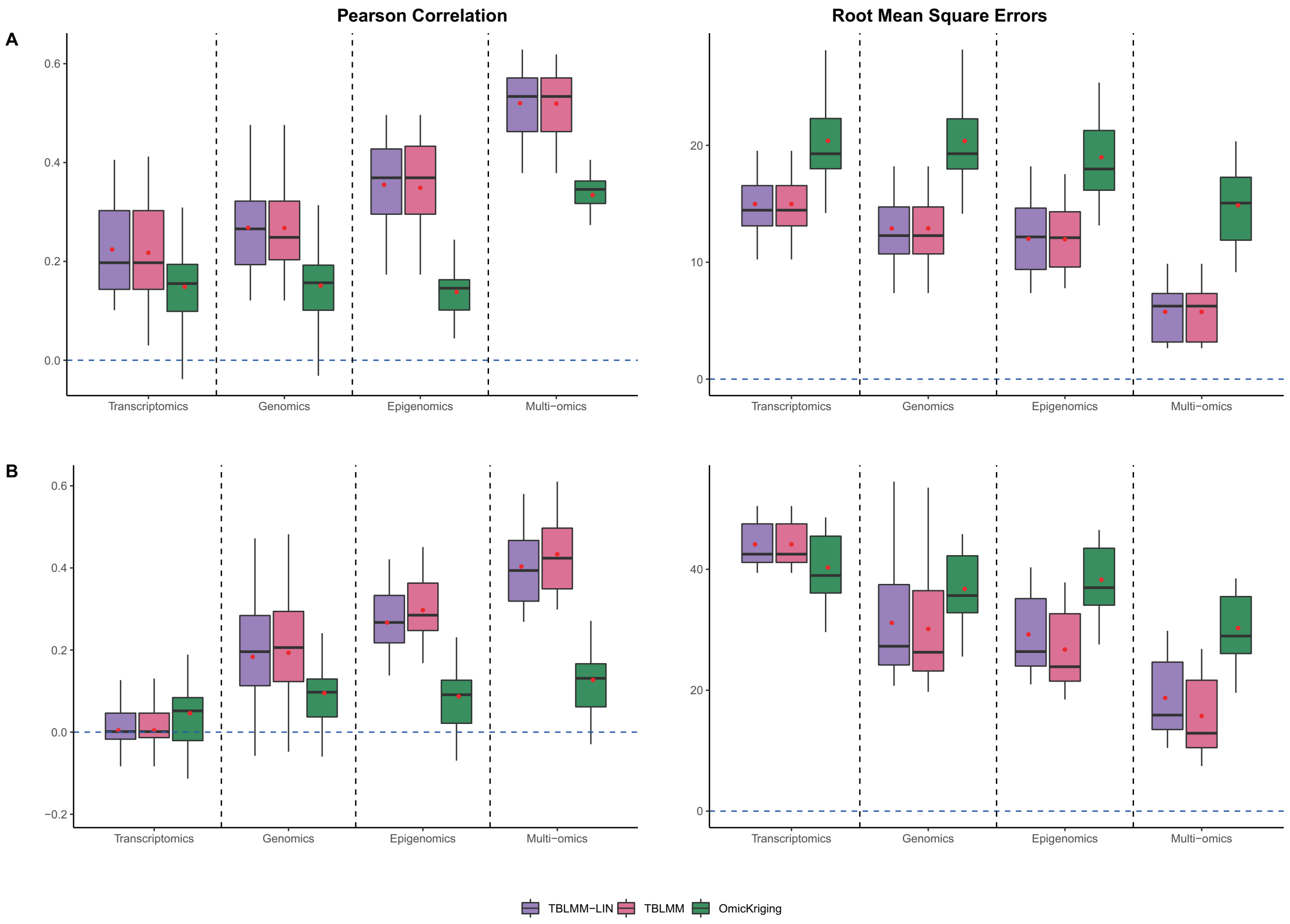

Fig. S4. The performance of TBLMM when the outcome is affected by multiple omics ($h^2 = 0.6$). Two settings were considered, including multiple omics data contribute to the outcomes independently (**A**) and only interactions between genomics and methylation contribute to the outcome (**B**).

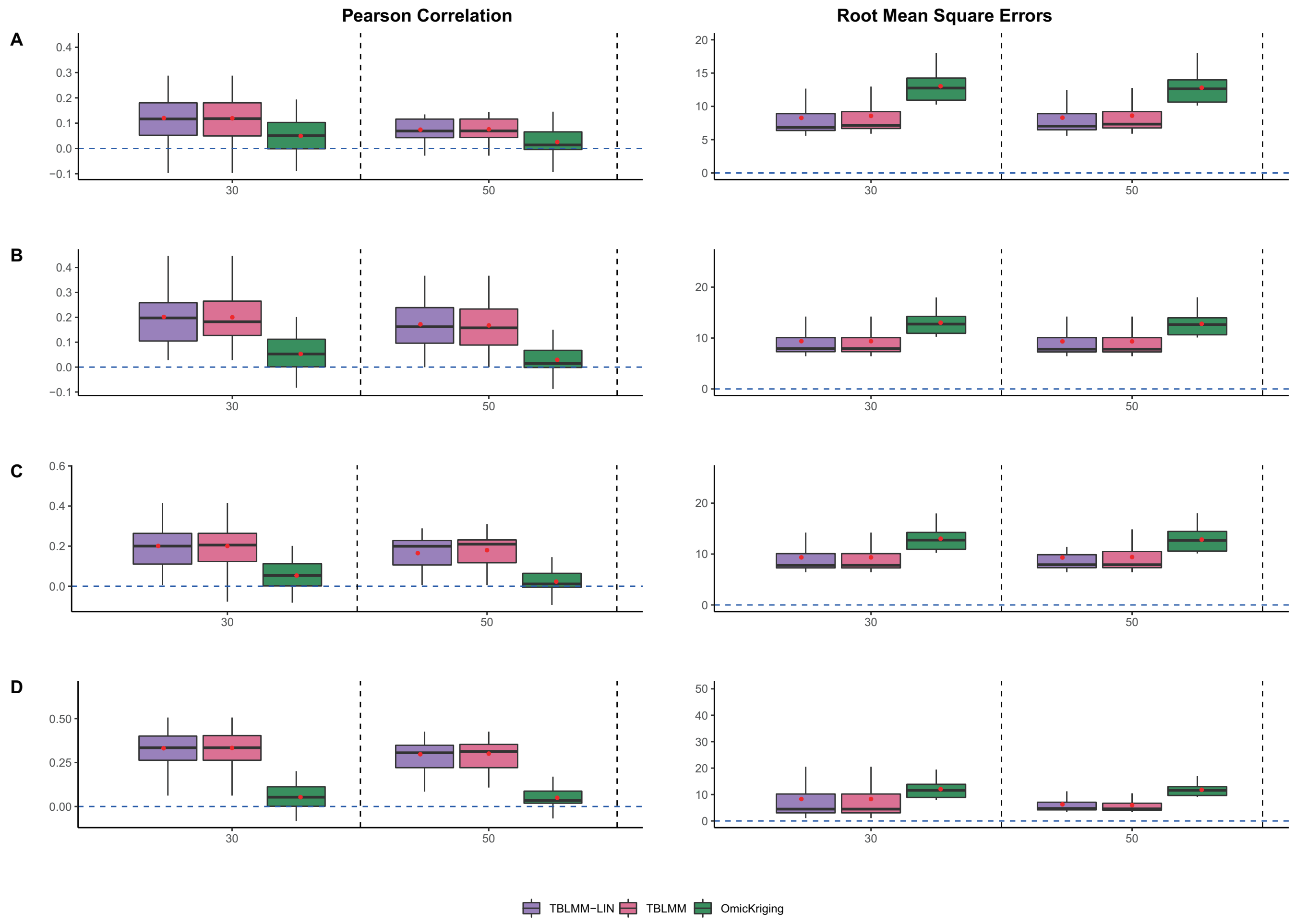

Fig. S5. The effects of the number of noise regions ($h^2 = 0.2$). The outcomes were modelled with only gene expression data (**A**), only genomics data (**B**), only methylation data (**C**), and all omics (**D**).

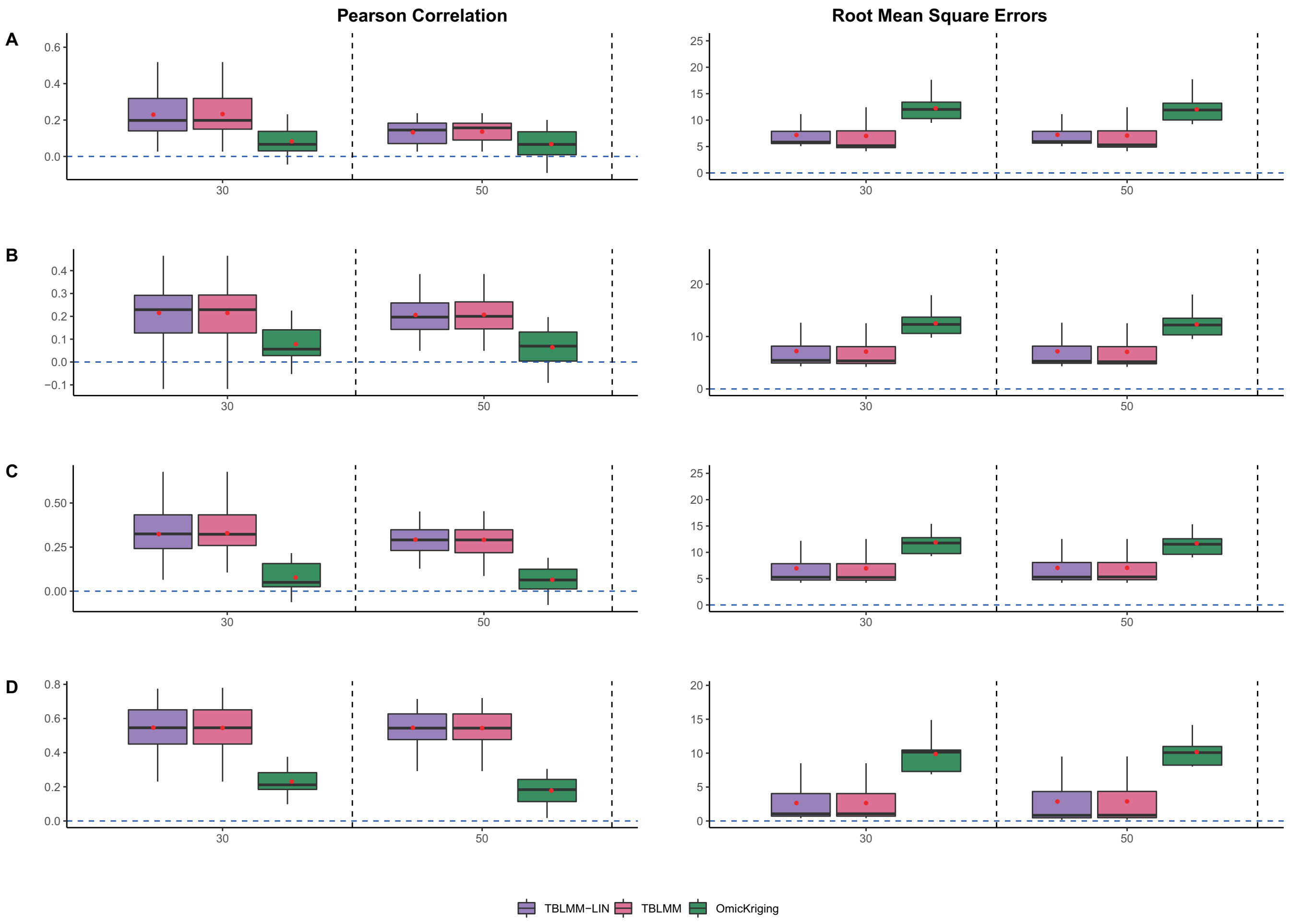

Fig. S6. The effects of the number of noise regions ($h^2 = 0.6$). The outcomes were modelled with only gene expression data (**A**), only genomics data (**B**), only methylation data (**C**), and all omics (**D**).

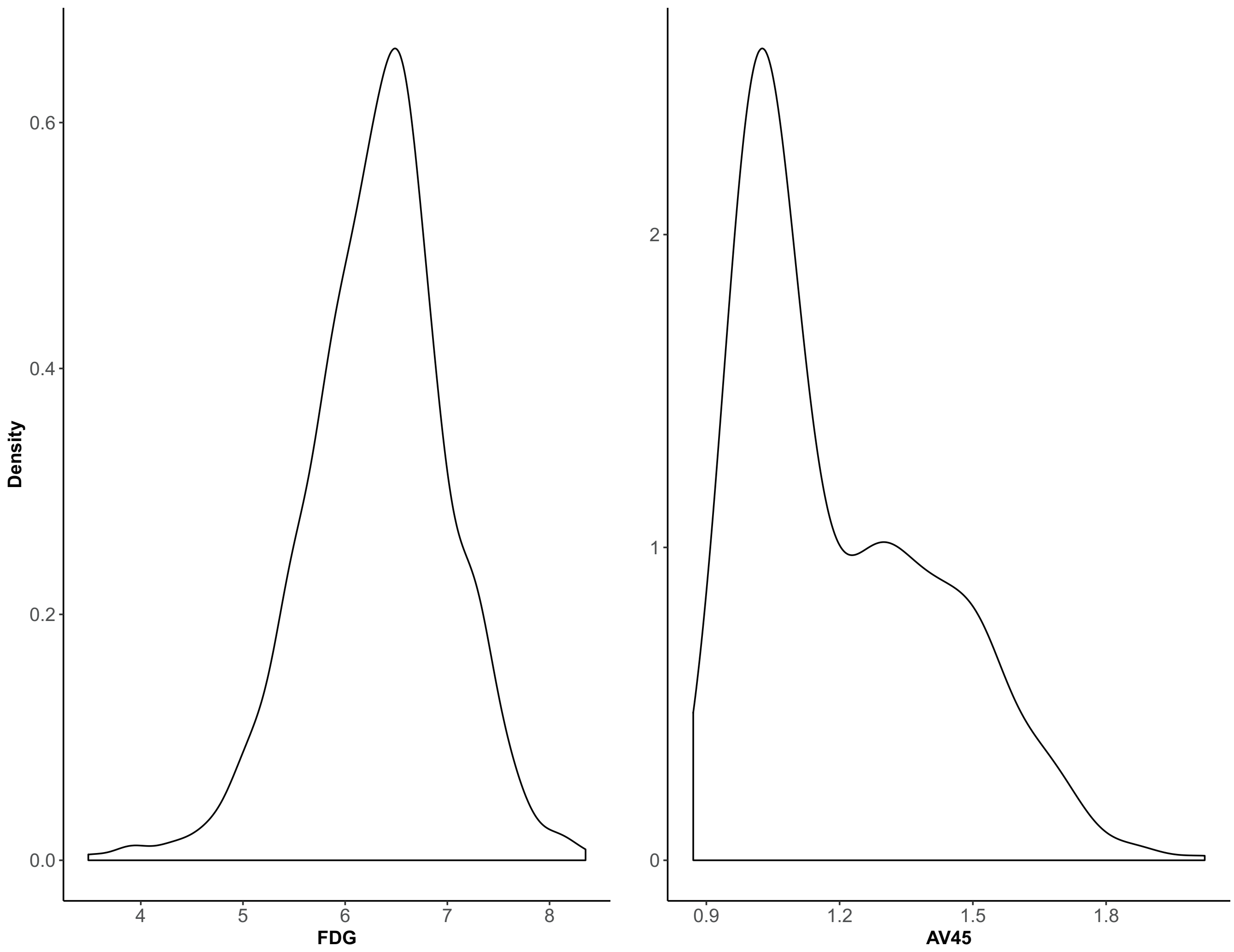

Fig. S7. Distributions for PET imaging data (FDG and AV45).

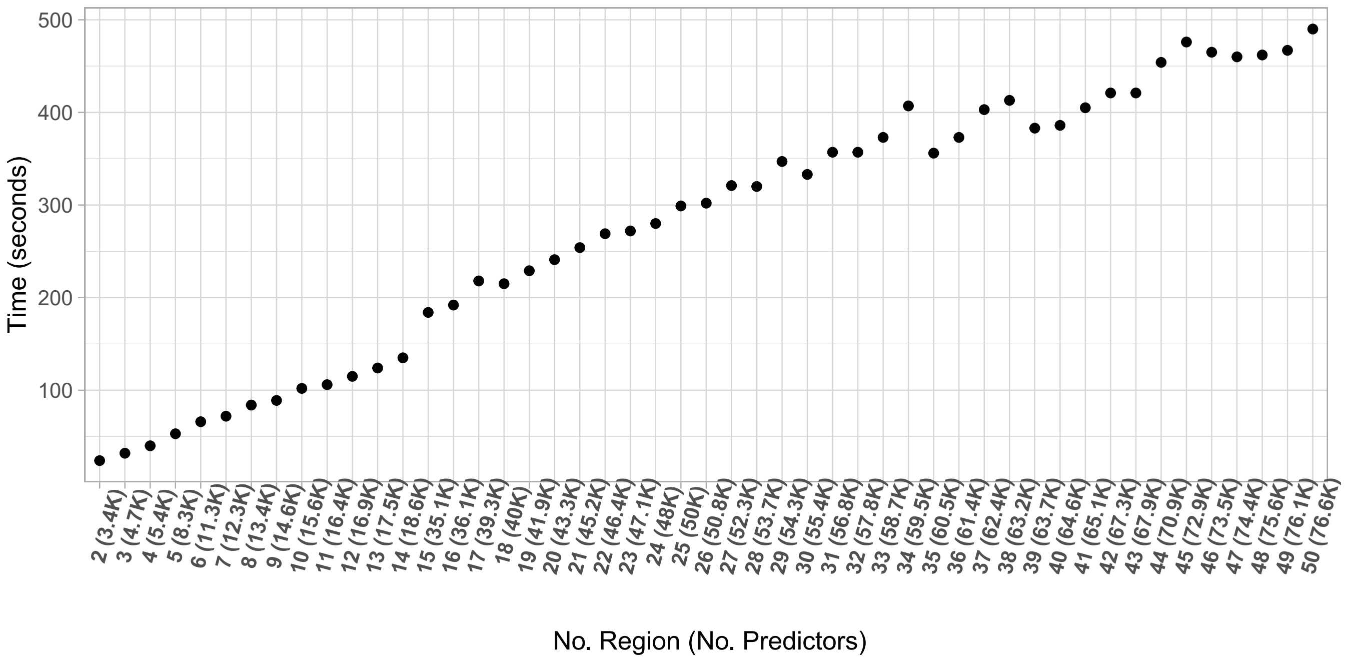

Fig. S8. The computational time as the number of predictors (regions) increases.
